# Editorial: Vaccination in children with immune-mediated diseases

**DOI:** 10.3389/fped.2023.1192407

**Published:** 2023-04-14

**Authors:** Natasa Toplak, Nicolaas M. Wulffraat, Yosef Uziel

**Affiliations:** ^1^ Department of Allergology, Rheumatology and Clinical Immunology, University Children’s Hospital, University Medical Centre Ljubljana, Ljubljana, Slovenia; ^2^ Faculty of Medicine, University of Ljubljana, Ljubljana, Slovenia; ^3^ Department of Pediatric Immunology & Rheumatology, Wilhelmina Children’s Hospital, University Medical Centre Utrecht, Utrecht, Netherlands; ^4^ Faculty of Medicine, Utrecht University, Utrecht, Netherlands; ^5^ Pediatric Rheumatology Unit, Department of Pediatrics, Meir Medical Center, Kfar Saba, Israel; ^6^ Sackler Faculty of Medicine, Tel Aviv University, Tel Aviv, Israel

**Keywords:** vaccination, paediatric rheumatic diseases, recommendations, vaccination completeness, vaccine hesitancy, COVID-19 vaccination

**Editorial on the Research Topic** Vaccination in children with immune-mediated diseases

Children with immune-mediated diseases comprise a large group of patients with rare diseases. The most common among them is juvenile idiopathic arthritis (JIA). Others, such as systemic lupus erythematosus (SLE), juvenile dermatomyositis, juvenile systemic scleroderma, and many diseases from the expanding group of autoinflammatory diseases affect less than one in 2000 people (1). Many of these patients are treated with immunomodulatory drugs, including conventional and biological disease-modifying antirheumatic drugs (DMARDs). The main adverse events of these drugs are infections. The most effective measure against infection remains vaccination; therefore, these children should be vaccinated when one is available. There are no major safety concerns for a vaccination with non-live vaccines, regardless of the therapy the children are receiving. However, caution is advised when live-attenuated vaccines are used in children treated with immunomodulatory suppressive therapy (2). Data on the safety of live-attenuated vaccines indicate that the small risk of infection with a vaccine strain is much lower than the risk of infection with a wild-type virus. Vaccination should also be considered for children treated with biological DMARDs (3).

Many cohort studies and a few RCTs were published on this topic, which was summarized in a systematic literature review for the 2021 update of the EULAR/PRES recommendations (Jansen et al.). The objectives of this paper are to update readers on advances in this field and to form the basis for clinical practice guidelines. These guidelines are increasingly used in efforts to standardize care across the globe. Here, PRES and CARRA collaborate with the new European reference networks (ERN). Guidelines are a clear priority for all ERNs. Pediatric rheumatology disorders fall under ERN RITA (www.ERN-RITA.org). Hopefully, they will be implemented in clinical practice. The literature review was the basis for a set of recommendations discussed and accepted by an international group of experts (2). Suffice it to say, in most instances, the existing national vaccination guidelines can be followed. It is important to note that due to the paucity of data on yellow fever vaccination, it is still contraindicated when under biological therapy.

With the new recommendations, one should not forget that the scientific community should also monitor whether they are integrated into clinical practice. Despite the clear evidence regarding the safety of measles, mumps, and rubella booster for patients treated with methotrexate, advice on its use for these cases is still subject to widespread concerns about its safety. Therefore, studies on vaccine coverage are very relevant, usually showing that coverage remains suboptimal (4). In the Swiss longitudinal, observational multicenter cohort study by Welzel et al. on vaccination completeness in children with rheumatic diseases, the overall vaccination rate in children with rheumatic diseases (PRD) was only 3.8% (Welzel et al.). However, for the vaccines included in the national immunization program vaccination, 70.1% were vaccinated. In a study by Balažiova et al. on real-life vaccination coverage in Slovak children with PRD, 117 of 156 patients (75%) were completely vaccinated (Balažiová et al.). Systemic immunosuppressive treatment was a significant predictor of incomplete vaccination coverage. These data need to be confirmed in other European countries, but they clearly illustrate that we need to strengthen our recommendations. Balažiova et al. advise close collaboration between pediatricians and rheumatologists to improve vaccination completeness.

Barriers to vaccination among children with childhood-onset SLE (c-SLE) and inflammatory bowel disease (IBD) were studied by Lloyd et al. In a cohort of 31 c-SLE and 26 IBD patients, vaccine hesitancy was not a barrier to vaccination. A needs-assessment survey among 30 primary care physicians (PCP) found that the obstacle to vaccination was discordance between parents and PCP comfort in vaccination. In the patient cohort, 93% felt that their PCP was well-informed about vaccination. However, only 40% of PCP felt very confident about vaccinating patients with these diseases.

Because of vaccine hesitancy among patients, parents, PCP, and sometimes even among pediatric rheumatologists, studies on the safety of vaccination in patients with PRD are of utmost importance. Especially in the time of the COVID-19 pandemic, when the first vaccine that was available for protection against SARS-CoV-2 infection was produced using a new mRNA technique, doubts about its safety were understandably even greater than usual. Efforts of several researchers soon produced evidence on the safety, immunogenicity, and efficacy of COVID-19 m-RNA vaccines (5–7). The Polish study that included 43 JIA patients, all vaccinated with the mRNA COVID-19 vaccine, showed no major adverse events after vaccination, and there was no connection between adverse events and the therapy the children were receiving. Disease activity remained stable after vaccination (Opoka-Winiarska et al.).

The article by Minoia et al. clearly demonstrated the hesitancy in pediatric vaccination when there is a paucity of data (Minoia et al.). They surveyed physicians (86% of whom were pediatric rheumatologists) regarding COVID-19 vaccination in patients who had multisystem inflammatory syndrome (MIS-C), and 290 replies from 236 centers in 61 countries were collected. The overall feeling was that the possible induction of MIS-C flares after SARS-CoV2 vaccination could be very serious. Thus, the consensus was to wait with this vaccine until 6 months after the MIS-C onset, which seems very wise.

Bizjak et al. discussed future challenges in vaccinology in pediatric rheumatology (Bizjak et al.). The review concluded that despite many new studies published in this area, especially those on live attenuated vaccines, there are still many unanswered questions. Long-term protection by vaccines, the need for booster doses, and the ideal time and timing of vaccination regarding the drug regimen are questions that need to be answered. Because IMDs are rare diseases, well-planned, multinational, multicenter, prospective studies are needed to achieve this goal.

The PRES vaccination study group is leading such a study.
